# Supplementary material for: Equilibrium Studies of Iron (III) Complexes with Either Pyrazine, Quinoxaline, or Phenazine and Their Catecholase Activity in Methanol
Source: Molecules. 2022 May 19;27(10):3257. doi: 10.3390/molecules27103257 (PMC9143456; doi:10.3390/molecules27103257)
Supplement: Supplementary file 1 [file molecules-27-03257-s001.zip › molecules-1696395-supplementary.pdf]

MATERIAL SUPPORTING TO

Equilibrium Studies of Iron(III) Complexes with either Pyrazine, Quinoxaline, or Phenazine and their Catecholase Activity in Methanol

José J. N. Segoviano-Garfias[1]*, Gabriela A. Zanor[1], Fidel Ávila-Ramos[1] and Egla Yareth Bivián-Castro[2]

[1] División de Ciencias de la Vida (DICIVA). Universidad de Guanajuato. Campus Irapuato-Salamanca. Ex Hacienda El Copal, Carretera Irapuato-Silao Km. 9, Irapuato, Gto. 36500 México.

[2] Centro Universitario de los Lagos. Universidad de Guadalajara. Enrique Díaz de León 1144, Col. Paseos de la Montaña 47460, Lagos de Moreno, Jalisco, México

*Corresponding author:

Tel: +524737405320

E-mail: segovi@ugto.mx

SUPPORTING INFORMATION FIGURES

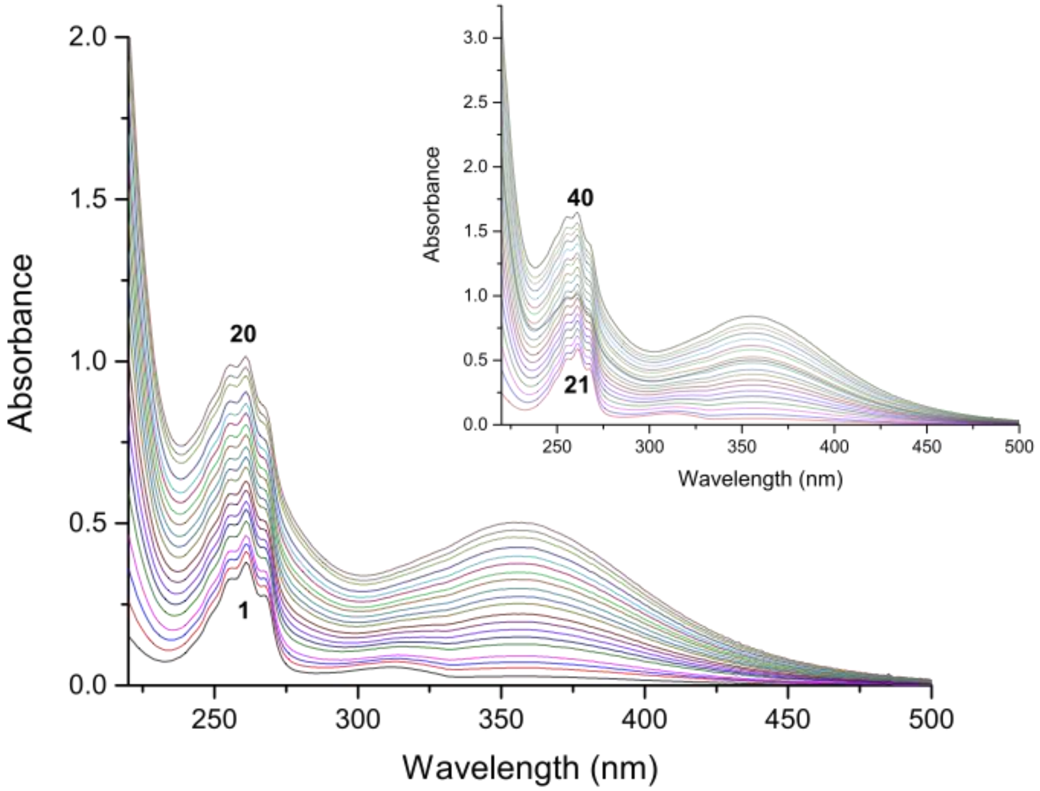

Figure S1. Absorption spectra of the iron(III)-pyrazine system in methanol solution: (a) For spectra 1-20, [pyrazine] = 50.0 µM and iron(III) concentration (µM): (1) 7.44; (2) 14.9; (3) 22.3; (4) 29.8; (5) 37.2; (6) 44.6; (7) 52.1; (8) 59.5; (9) 67.0; (10) 74.4; (11) 81.8; (12) 89.3; (13) 96.7; (14) 104.0; (15) 112.0 ; (16) 119.0; (17) 126.0; (18) 134.0; (19) 141.0; (20) 149.0. For spectra 21-40, [pyrazine] = 75.0 µM and iron(III) concentration (µM): (21) 11.2; (22) 22.3; (23) 33.5; (24) 44.6; (25) 55.8; (26) 67.0; (27) 78.1; (28) 89.3; (29) 100.0; (30) 112.0; (31) 123.0; (32) 134.0. (33) 145.0; (34) 156.0; (35) 167.0; (36) 179.0; (37) 190.0; (38) 201.0; (39) 212.0; (40) 223.0.

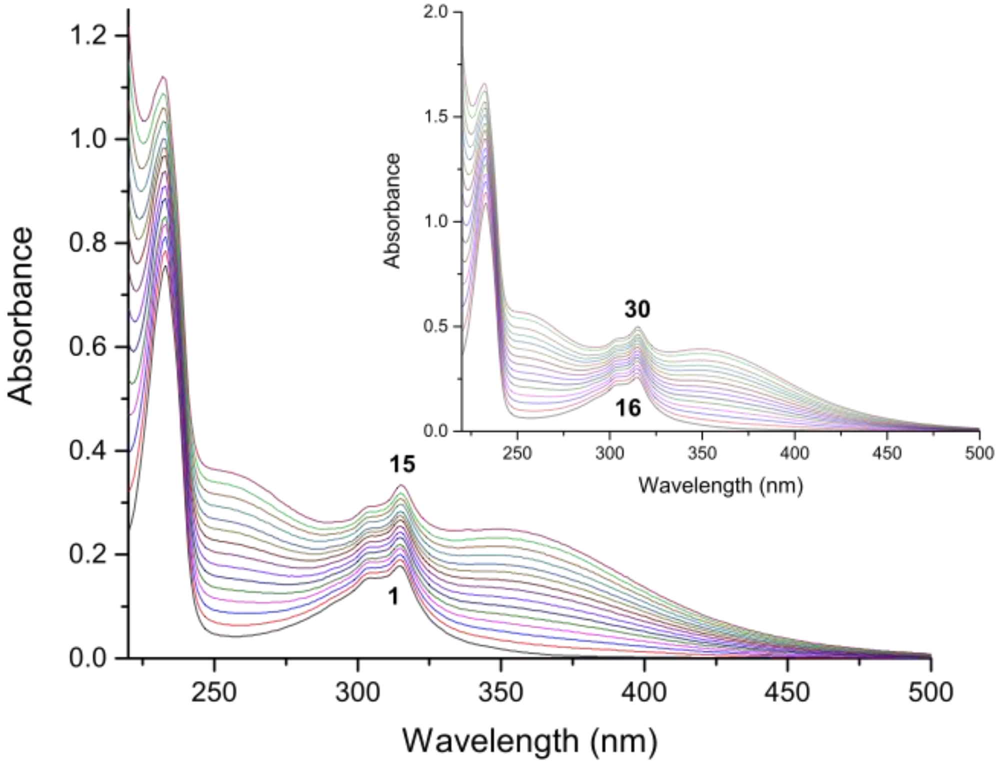

Figure S2. Absorption spectra of the iron(III)-quinoxaline system in methanol solution: (a) For spectra 1-15, [pyrazine] = 24.6 μM and iron(III) concentration (μM): (1) 2.57; (2) 7.71; (3) 12.9; (4) 18.0; (5) 23.1; (6) 28.3; (7) 33.4; (8) 38.6; (9) 43.7; (10) 48.8; (11) 54.0; (12) 59.1; (13) 64.3; (14) 69.4; (15) 74.5. For spectra 16-30, [quinoxaline] = 36.9 μM and iron(III) concentration (μM): (16) 3.96; (17) 11.9; (18) 19.8; (19) 27.7; (20) 35.6; (21) 43.6; (22) 51.5; (23) 59.4; (24) 67.3; (25) 75.2; (26) 83.2; (27) 91.1; (28) 99.0; (29) 107.0; (30) 115.0.

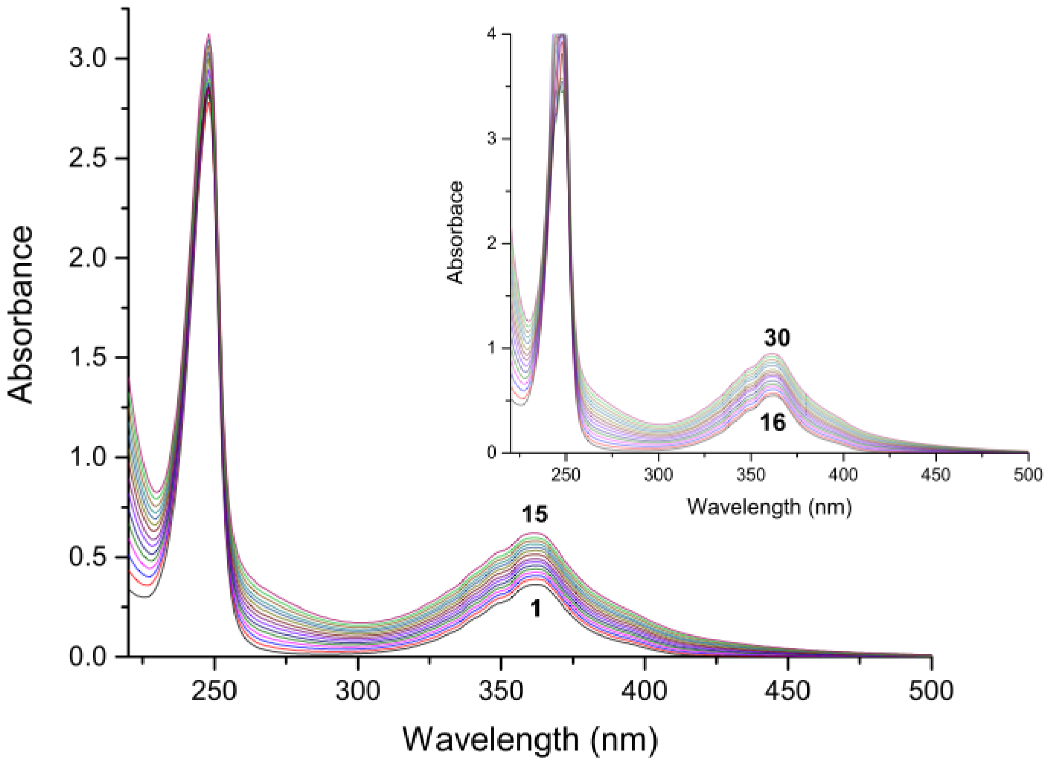

Figure S3. Absorption spectra of the iron(III)-phenazine system in methanol solution: (a) For spectra 1-15, [phenazine] = 24.6 µM and iron(III) concentration (µM): (1) 2.57; (2) 7.71; (3) 12.9; (4) 18.0; (5) 23.1; (6) 28.3; (7) 33.4; (8) 38.6; (9) 43.7; (10) 48.8; (11) 54.4; (12) 59.1; (13) 64.3; (14) 69.4; (15) 74.5. For spectra 16-30, [phenazine] = 36.9 µM and iron(III) concentration (µM): (16) 3.96; (17) 11.9; (17) 19.8; (19) 27.7; (20) 35.6; (21) 43.6; (22) 51.5; (23) 59.4; (24) 67.3; (25) 75.2; (26) 83.2; (27) 91.1; (28) 99.0; (29) 107.0; (30) 115.0.

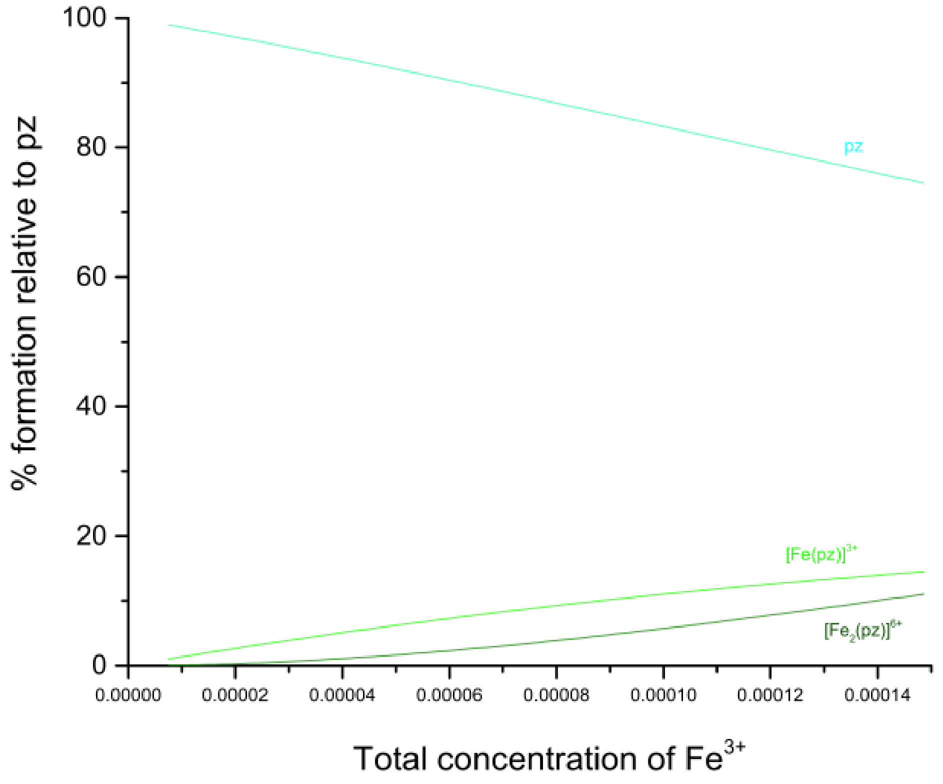

Figure S4. Formation curves of the iron(III)-pyrazine complexes in methanol. [pz]= 50 µM and $[Fe]^{3+}$ range from 7.44 to 148.8 µM.

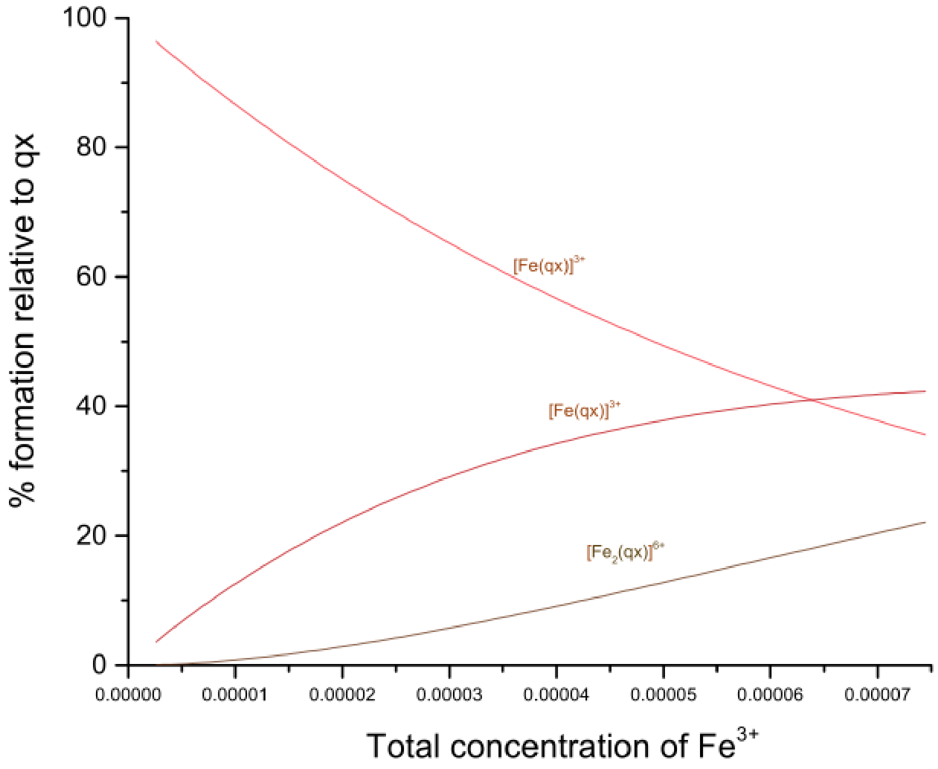

Figure S5. Formation curves of the iron(III)-quinoxaline complexes in methanol. [qx]= 24.85 μM and $[Fe]^{3+}$ range from 2.57 to 74.53 μM.

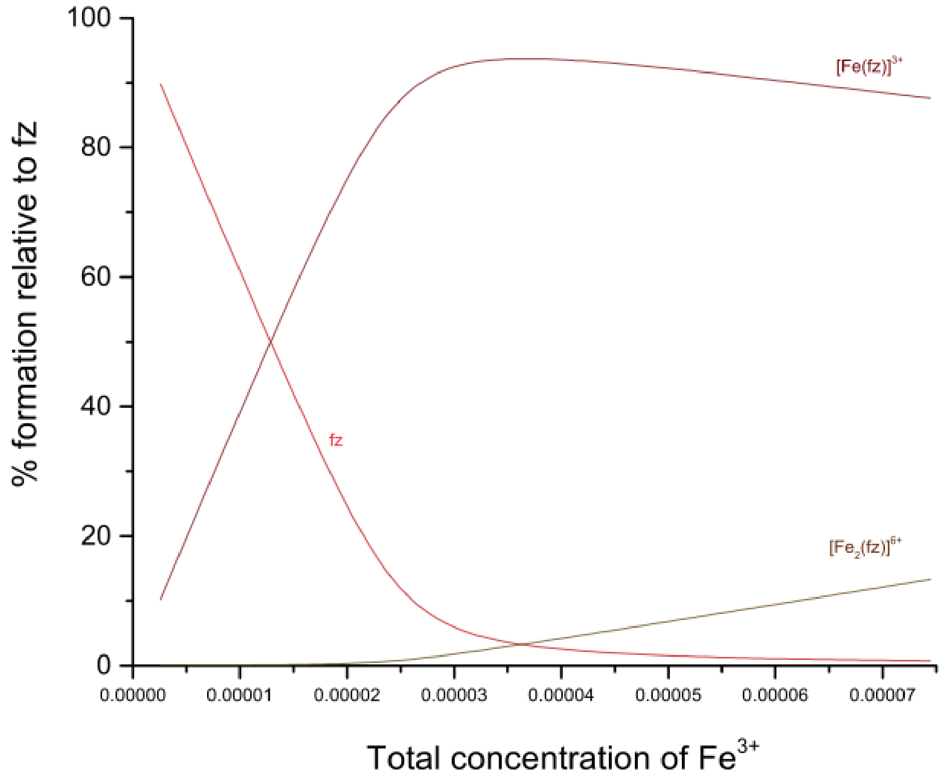

Figure S6. Formation curves of the iron(III)-phenazine complexes in methanol. [fz]= 24.85 µM and $[Fe]^{3+}$ range from 2.57 to 74.53 µM.

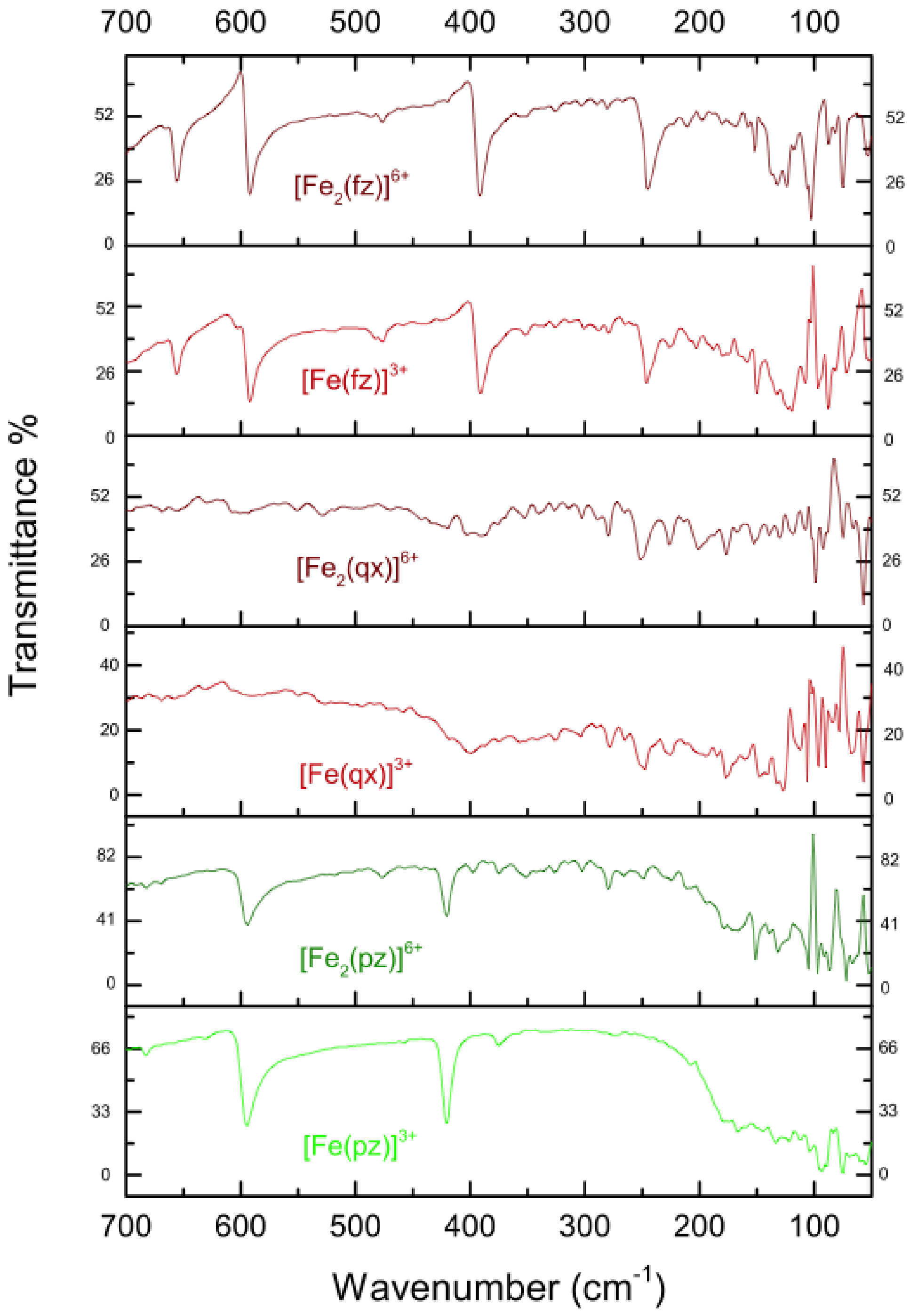

Figure S7. Far-infrared spectra of the different iron(III) complexes obtained in this study: $[Fe(pz)]^{3+}$, $[Fe(qx)]^{3+}$, $[Fe(fz)]^{3+}$,$[Fe_2(pz)]^{6+}$, $[Fe_2(qx)]^{6+}$ and $[Fe_2(fz)]^{6+}$.

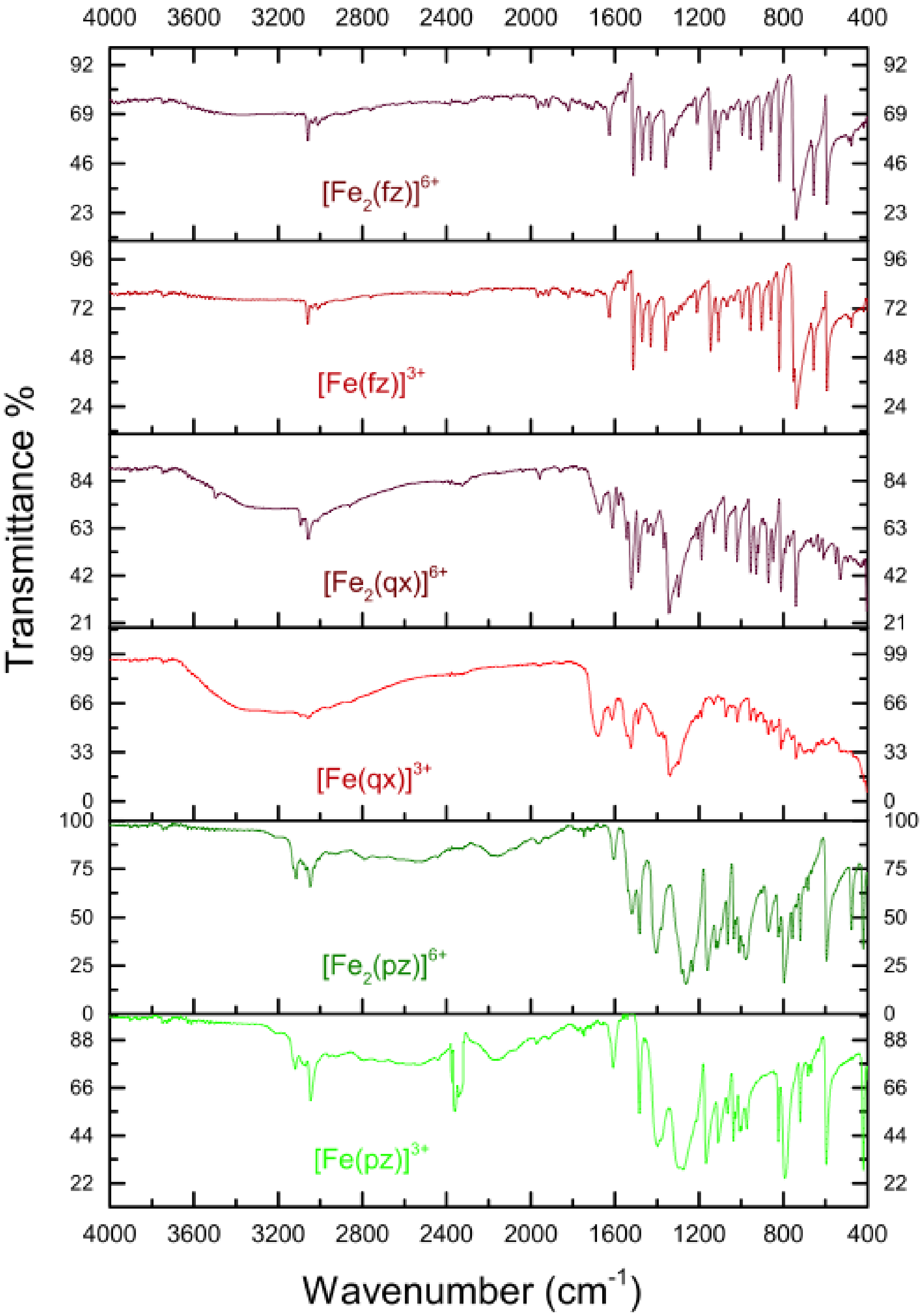

Figure S8. Mid-infrared spectra of the different iron(III) complexes obtained in this study: $[Fe(pz)]^{3+}$, $[Fe(qx)]^{3+}$, $[Fe(fz)]^{3+}$,$[Fe_2(pz)]^{6+}$, $[Fe_2(qx)]^{6+}$ and $[Fe_2(fz)]^{6+}$.

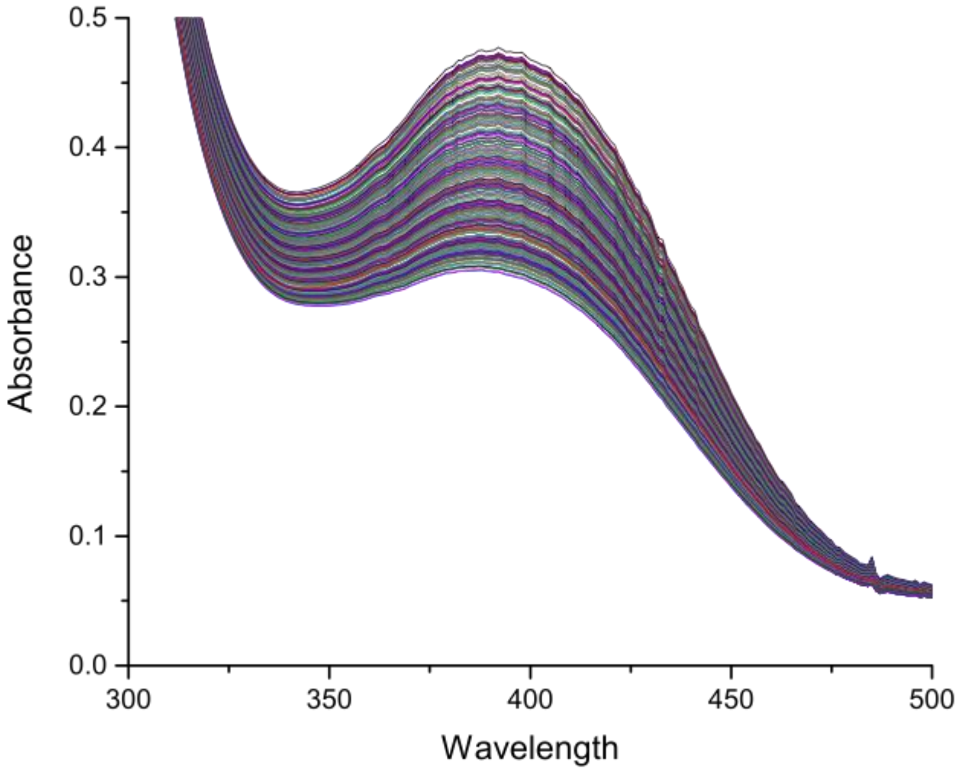

Figure S9. Typical oxidation reaction of the 3,5-di-tert-butyl catechol (DTBC) to 3,5-di-tert-butyl-*o*-benzoquinone (DTBQ) monitored at 400 nm.
